# Supplementary material for: Yiai Fuzheng decoction inhibits triple-negative breast cancer by remodeling the immune microenvironment
Source: Front Immunol. 2025 Sep 30;16:1615631. doi: 10.3389/fimmu.2025.1615631 (PMC12518410; doi:10.3389/fimmu.2025.1615631)
Supplement: Supplementary file 2 [file DataSheet1.zip › Supplementary File 2/Treatment/C_vs_D_vs_H/02_PCA/All_Groups/All_Combinaiton/PC13_Score_2D_Label.pdf]

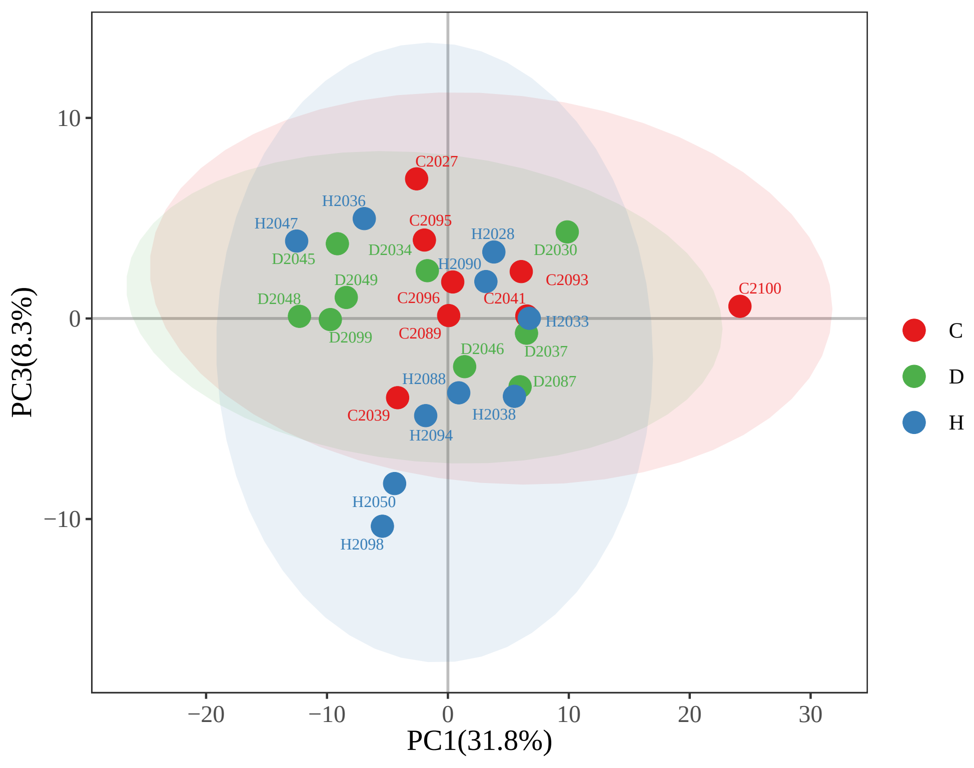
C2027
H2036
H2047
C2095
H2028
D2030
D2045
D2034
H2090
C2093
D2049
C2096
C2041
C2100
D2048
H2033
D2099
C2089
D2037
D2046
H2088
D2087
C2039
H2038
H2094
H2050
H2098
10
0
−10
−20
−10
0
10
20
30
PC1(31.8%)
PC3(8.3%)
C
D
H
